# Commentary: Differential Cerebral Response to Somatosensory Stimulation of an Acupuncture Point vs. Two Non-Acupuncture Points Measured with EEG and fMRI

**DOI:** 10.3389/fnhum.2016.00063

**Published:** 2016-02-19

**Authors:** Yiu Ming Wong

**Affiliations:** ^1^Health Science Unit (PEC), Hong Kong Physically Handicapped and Able Bodied Association, Kowloon, Hong Kong

**Keywords:** fMRI, acupuncture, human anatomy, somatosensory stimulation, functional connectivity

Acupuncture is a traditional form of Asian medicine; the practice involves needling the invisible lines, called meridians, on the skin’s surface that have acupuncture points within and run longitudinally up and down the human body. However, modern anatomy does not well support the existence of meridians and acupuncture (Litscher, 2014). On the other hand, some researchers have asserted that acupuncture point stimulation caused human somatosensory cortex responses being detected in an fMRI. This phenomenon has been regarded as indirect evidence of the specificity of acupuncture points, although the evidences are debatable (Cho et al., 1998, 2006). In a recent article by Nierhaus et al. (2015) that evaluated acupuncture-induced brain responses using both an EEG and an fMRI, the authors concluded that the needles inserted into a known acupuncture point, named ST36, led to different EEG and fMRI patterns as compared to the needling of two non-acupuncture points. I am expressing my opinions on the choice of the non-acupuncture point labeled Control Point 1 (CP1) in the above article.

## Do ST36 and CP1 Share the Same Dermatome?

**Figure 1:**
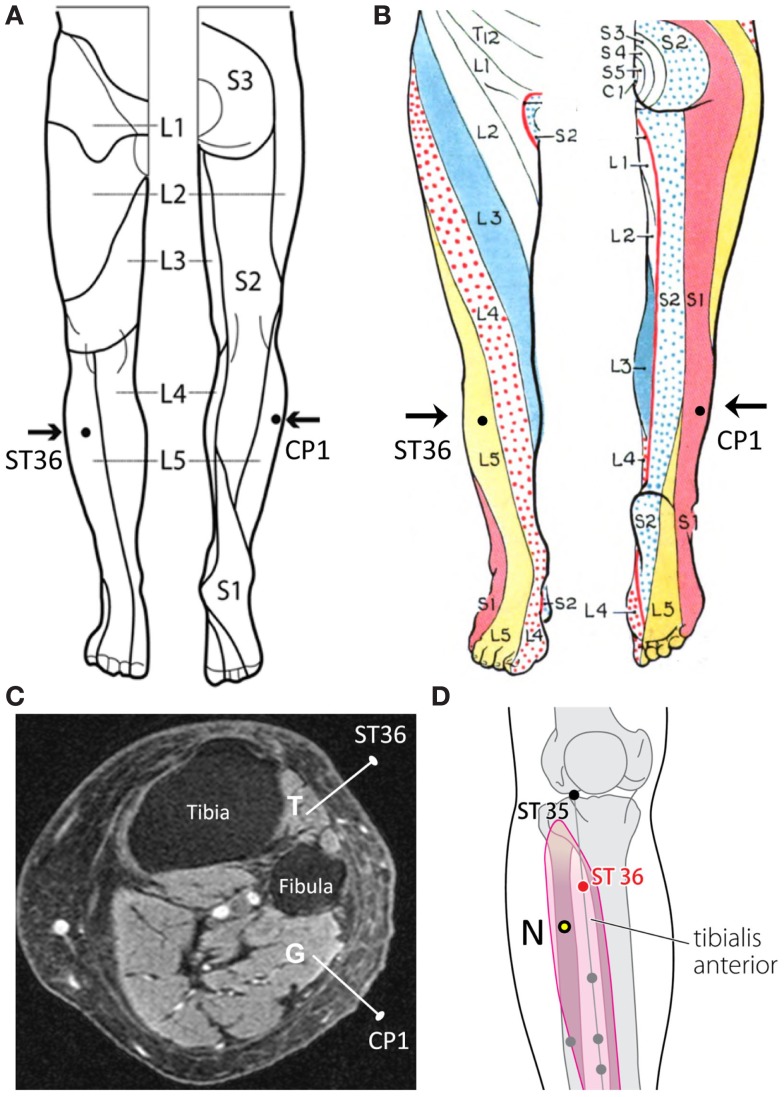
**(A)** ST36 and CP1 placements located within the dermatome L5 in Nierhaus et al. (2015). **(B)** ST36 and CP1 placements located in different dermatomes according to Grant (1972). **(C)** Axial MRI of the right lower leg that illustrates the tibialis anterior (T) and gastrocnemius (G) with needles inserted. **(D)** N = suggested CP1 that is not a known acupuncture point.

The basic concept of dermatomes is that the area of skin innervated by an individual spinal cord segment is referred to as a dermatome. In order to prove the theory that the specificity of the ST36 point to the brain responses vs. that of the CP1 point, the authors asserted that both ST36 and CP1 are within the same dermatome region of L5 (Figure 1A). However, the authors’ assertion may be oversimplified as an overlap of innervations between adjacent dermatomes is common and leads to variability between an individuals’ dermatome distributions (Downs and Laporte, 2011). In addition, striking inconsistencies within dermatome maps have been found in standard anatomy and medical textbooks (Lee et al., 2008). For example, if the dermatome map of Grant (1972) were used, the ST36 and CP1 points would be located in dermatomes of L5 and S1, respectively (Figure 1B).

## Different Tissues Under ST36 and CP1 not Well Mentioned

The authors stated that the needle was vertically inserted 1–2 cm deep into the skin areas of ST36 and CP1. The muscle beneath the ST36 point is tibialis anterior, which is innervated by deep peroneal nerve (L4, L5, and S1); under the CP1 point is the lateral gastrocnemius, which is innervated by tibial nerve (S1 and S2) (Figure 1C). Because the ST36 and CP1 points have distinct muscular and neural components, segmental innervations, and corresponding cerebral cortex (Dobkin et al., 2004), this is not strong evidence for the theory that the ancient acupuncture point is the unique explanation for the fMRI and EEG patterns. In other words, Nierhaus et al.’s (2015) choice of CP1 may influence their interpretations on the specificity of ST36 relevant to the brain activities.

This reasoning may be supported by Kawczyński et al. (2013) preliminary data that the skin areas of ST36 and CP1 had incomparable pain pressure thresholds. Theoretically, these two areas given the identical stimulus may lead to incomparable EEG patterns (Chien et al., 2014).

## Suggestions

Acupuncture-induced brain activity in somatosensory cortex areas would be expected from a variety of sensory inputs, such as cutaneous layer of proposed acupuncture point, subcutaneous and muscular layers underneath, and different nerve endings in these three layers. As such, concern is advised when producing acupuncture-related studies using fMRI and EEG, in which the known acupuncture and non-acupuncture points should share the same dermatome and myotome, and have comparable tactile sensitivity. Thus, I would suggest that the CP1 could be placed posterior and inferior to the ST36 (Figure 1D), in which the real and control needling would penetrate the same muscle as well as the same dermatome; this suggested CP1 has been confirmed as a non-acupuncture point (WHO Regional Office for the Western Pacific, 2008).

## Author Contributions

This is to prove that the sole author named above participated in drafting the article and revising it critically for important intellectual content, and the author gave final approval of the version to be submitted and any revised version.

## Conflict of Interest Statement

The author declares that the research was conducted in the absence of any commercial or financial relationships that could be construed as a potential conflict of interest.
